# How to Unmask Hidden Cardiovascular Diseases through Preparticipation Screening in Master Athletes?

**DOI:** 10.31083/j.rcm2312405

**Published:** 2022-12-12

**Authors:** Kinga Zujko, Łukasz A. Małek

**Affiliations:** ^1^Department of Cardiology, Medical University of Bialystok, 15-276 Bialystok, Poland; ^2^Department of Epidemiology, Cardiovascular Disease Prevention and Health Promotion, National Institute of Cardiology, 04-635 Warsaw, Poland

**Keywords:** cardiovascular disease, hidden hypertension, cardiovascular risk factors, sudden cardiac death, master athletes, endurance training, preparticipation screening, prevention, athlete's heart

## Abstract

Cardiovascular disease (CVD) is the most common cause of death globally in general population. Sport activity is an effective and recommended non-pharmacological method of CVD prevention. Presently, the group of people practicing sport regularly is constantly growing due to increasing awareness of its health benefits. However, vigorous-intensity exercises can reveal previously undetected disease. Master athletes over 35 years old are particularly exposed to sudden cardiac death (SCD) mainly in the course of coronary artery disease (CAD). Another common disease in veteran athletes is hypertension. It is known that regular endurance training can lower blood pressure at rest, so arterial hypertension in athletes is usually masked by adaptation to effort. Despite of normal or high-normal blood pressure in the office, the values during exercises and in ambulatory blood pressure monitoring (ABPM) can exceed the norm. Hidden hypertension have the same negative impact on cardiovascular system. It increases the risk of (1) atherosclerosis and therefore myocardial infarction or stroke, (2) left ventricular hypertrophy with diastolic and/or systolic heart failure, myocardial fibrosis and ventricular arrhythmias, (3) left atrial enlargement increasing the risk of atrial fibrillation and stroke and (4) aortic dilation/dissection. Through these complications hypertension can lead to SCD during sport activities, therefore it is important to recognize this disease early and start a proper treatment. To enable safe participation in sports competition detailed guidelines for screening were created, but they mainly concern CAD. We propose an additional scheme of screening in master athletes including the detection of hidden hypertension to prevent its consequences.

## 1. Introduction 

Cardiovascular diseases are still leading cause of death globally (WHO), among which ischemic heart disease is the most common. Presently people are more aware of benefits of physical activity. Current WHO guidelines recommend 150–300 minutes of moderate (40–69% peak oxygen uptake—VO2 max, 55–74% maximal heart rate—HR max) or 75–150 minutes of intensive (70–85% VO2 max, 75–90% HR max) aerobic exercises per week [1]. Physically active individuals have lower risk of ischemic cardiovascular disease, stroke and mortality compared to a cohort with a sedentary lifestyle [2]. As a result not only young, but also middle-aged and elderly people want to improve their physical capabilities and participate in competitions. Master (veteran) athletes represent group of athletes in an age category over 35 years, who compete in endurance sports events including for example athletics, swimming, cycling or combination of those. Veteran sports exist from many years and even World Masters Athletics Championship began already in 1975. However, the group of over 35 years old includes a variety of athletes—those who are continuously training since childhood, athletes who want to return to training after a long break, but also a growing number of those who never had contact with competitive sport before. We wanted to highlight the most important issues related to cardiovascular risk in the latter group of veteran athletes, who might have never undergone preparticipation medical screening.

## 2. Cardiovascular Risk in Amateur Master Athletes

Despite of benefits from regular training, an instant load of huge effort can result in dangerous adverse events, especially when it concerns insufficiently trained people who began to participate in competitive sport and may have already developed acquired cardiovascular diseases such as the amateur veteran athletes. Many of those individuals had never undergone cardiovascular system assessment before they start to compete in sports events. It poses them at risk of sudden cardiac death (SCD) during exercise, which is determined as unexpected death due to cardiac causes that occur within 1 hour of the onset of symptoms. Risk of SCD is increased in low-trained athletes and most of incidents happen in individuals over 35 years old during recreational activity [3, 4]. The cardiovascular conditions which can lead to SCD are specific for each age group. In young athletes dominate coronary artery anomalies and arrhythmias mainly due to congenital diseases. In age <35 years old even morphologically normal heart does not eliminate the risk of SCD [5], because of ion-channelopathies recognizable in electrocardiogram. For master athletes most common is coronary artery disease (CAD)and myocardial diseases such as left ventricular hypertrophy and fibrosis potentially caused by overt or hidden hypertension as well as cardiomyopathies—arrhythmogenic cardiomyopathy (ACM), hypertrophic cardiomyopathy (HCM), dilated cardiomyopathy (DCM) or myocarditis [6, 7]. The direct cause of death usually, apart from aortic dissection, is ventricular arrhythmia, occurring both in the course of structural heart disease and without prior medical history. It is triggered by elevated level of catecholamines and potassium as well as by increased sympathetic system stimulation observed during intensive exercise. For several minutes after the end of activity, the concentration of catecholamines continues to increase, while a sudden decrease in potassium concentration is noted [8]. The presence of cardiac ischemia, dehydration, overheating and/or other electrolyte imbalance during or after the intense exercise can further exacerbate arrhythmogenic influence of the factors mentioned above. Additionally, some of SCD in athletes can occur at rest [6]. Former male endurance athletes have also higher risk of atrial fibrillation and bradyarrhythmias and their frequency is associated with number of completed races and their finishing time [9].

## 3. Most Common Cardiovascular Risk Factors in Master Athletes

### 3.1 Overt or Masked Hypertension

During endurance physical activity cardiac output increases due to the multiplied stroke volume with an effort up to 30% VO2 max and then due to constantly increasing heart rate. It is paralleled by rise of the mean arterial pressure (MAP), but mainly because of increasing systolic blood pressure (SBP). SBP should not exceed 210 mmHg in men or 190 mmHg in women during maximal exercise and diastolic blood pressure (DBP) should nor rise ≥105 mmHg [10]. Resistance sports are associated with even higher SBP increase. On the other hand, regular repetitive physical activity, especially endurance effort, reduces heart rate and blood pressure at rest, what is protective for vessel walls [11, 12, 13].

Arterial hypertension defined as a blood pressure values ≥140/90 mmHg is one of the most common chronic disease in general population and also in master athletes [14, 15]. The first step in master athletes with a newly diagnosed hypertension is a non-pharmacological treatment consisting of lifestyle changes such as healthy diet, body mass reduction and quitting smoking. It is recommended that people with arterial hypertension engage in at least 30 minutes of moderate aerobic exercise for 5–7 days/week what could reduce SBP by 7 mmHg and DBP by 5 mmHg [16, 17]. Furthermore, resistance training is also effective in reducing BP at rest and is recommended for 2–3 days/week in addition to aerobic training [17]. Ambulatory blood pressure monitoring (ABPM) should be performed when there is no improvement after 3–6 months and BP is still ≥140/90 mmHg to exclude white coat syndrome [18]. Lifestyle changes and reduction of risk factors are required in all grades of hypertension. Pharmacotherapy is needed immediately after twice repeated BP values ≥160/100 mmHg or BP ≥180/110 mmHg which does not need to be confirmed at the next visit and is enough to implement treatment [16]. The most desirable feature of hypertension drug for athletes is the lack of effect on athletic performance, therefore angiotensin-converting enzyme inhibitors and angiotensin II-receptor blockers are preferred [19]. Furthermore, renin-angiotensin system disturbances predominate in the pathophysiology of hypertension [20]. Another choice in the pharmacotherapy are calcium channel blockers and alpha adrenergic blocking agents. Beta-blockers can have a negative impact on physical performance and are forbidden in precision sports such as archery, shooting and fencing. World Anti-Doping Agency does not allow diuretics in all competitive sports [21]. Hypertension stage 1 is not a contraindication to training, but all athletes should undergo echocardiography to assess possible cardiac complications of this chronic disease. Left ventricular hypertrophy and other signs of a target organ damage are an indication to suspend participation in competitive sports until proper blood pressure is achieved. Athletes with severe hypertension should avoid strenuous physical exertion until they reach optimized BP values.

In some cases adaptive response of heart to exercises can mask hypertension. Hidden hypertension may be suspected if through regular repetitive training athletes have normal blood pressure in the office and at home, but ABPM reveals mean 24 hours’ values ≥130/80 mmHg [13]. Both regular intensive physical activity and hypertension lead to remodeling of the heart. Response to endurance training as an effect of increased preload leads to eccentric hypertrophy, characterized by left and right ventricle end-diastolic volume increase and bi-atrial enlargement with or without mild wall thickening and an increased early diastolic filling [22, 23]. Masked hypertension leads to greater arterial stiffness measured with carotid-femoral pulse wave velocity (PWV) [24], but this measurement is more associated with higher pulse pressure (PP) [25]. We have recently found that a mild left ventricular hypertrophy (LVH) can be considered as a marker of a masked hypertension. Masked hypertension was found in 70% of master athletes with LVH and in 37% of those without LVH despite normal office and home BP [26].

**Fig. 1.**
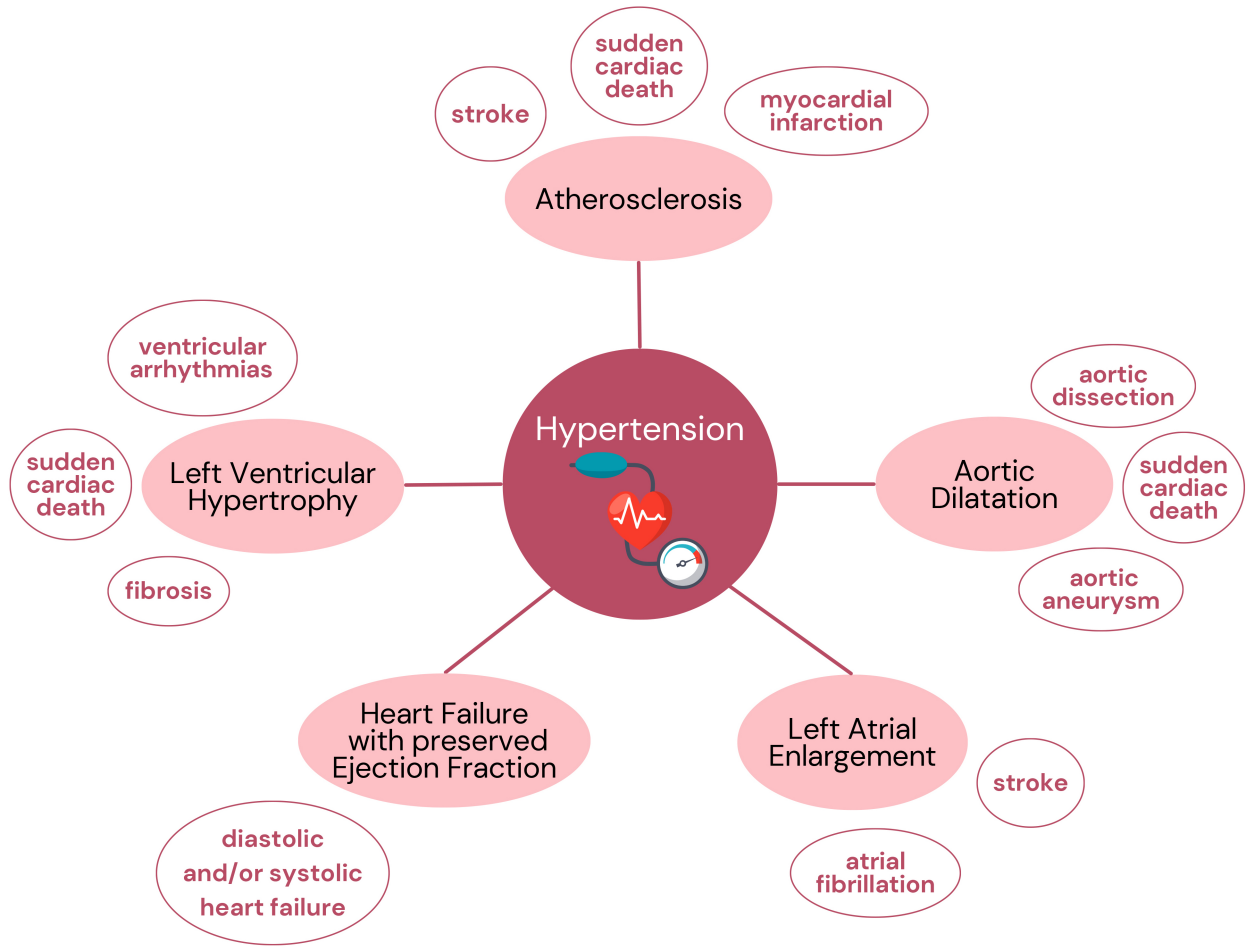
**Complications of uncontrolled hypertension**.

Hypertension masked by exercise is more difficult to detect, but has the same consequences (Fig. 1). It increases the risk of cardiovascular events and causes destruction of internal organs such as heart failure and renal dysfunction [27, 28]. Hypertensive response to exercise is also associated with higher risk of cardiovascular events and mortality [29], so it is important to detect hidden hypertension early in order to start right treatment and prevent complications.

### 3.2 Coronary Artery Disease

The other common medical condition which can be found in master-athletesis coronary artery disease (CAD) [30], mainly caused by ongoing atherosclerosis. Additionally, chronic coronary syndrome (CCS) is the most common cause of SCD in athletes over 35 years old [5, 7]. This condition requires special attention, because of its asymptomatic course for many years. Gradually progressing occlusion of vessels can lead to critical occlusion or plaque rapture and cause acute coronary syndromes (ACS) with all its consequences. Hypertension is strongly associated with CAD by causing vascular endothelial damage, which induces atherosclerosis [31, 32]. Other risk factors that increase the probability of atherosclerotic plaque progression are age, male gender, non-high-density lipoprotein (non-HDL) cholesterol level, smoking, alcohol, diabetes, obesity and sedentary lifestyle [33]. Management options in CAD prevention and treatment include lifestyle modification, pharmacological treatment, revascularization and proper risk rate and effort selection. Physical activity improves triglycerides level, but only mildly affects low-density lipoprotein (LDL) levels and when necessary treatment with lipid lowering drugs should be initiated [34]. Competitive or intensive efforts can reveal symptoms of angina and result in cardiac ischemia and rhythm disturbances [35].

### 3.3 Left Ventricular Hypertrophy and Heart Failure

As previously mentioned, major cause of SCD in athletes over 35 years old is left ventricular hypertrophy (LVH) [6] with HCM being the main cause, followed by hypertension. It is also possible that some cases of the so-called idiopathic LVH may be caused by undiagnosed or masked hypertension. While eccentric hypertrophy without diastolic impairment is considered to be the result of endurance exercise, concentric hypertrophy with relaxation disturbances could be an effect of hidden or already diagnosed but improperly treated hypertension [22, 23]. Athletes with higher blood pressure have not only higher left ventricular mass/volume ratio and LV wall thickness, but also a lower diastolic function [24, 34]. In some athletes lower left ventricle function can occur at rest, but if it is a physiological phenomenon it should improve during stress echocardiography by at least 13% contrary to prolonged systolic dysfunction observed in hypertension [36]. To differentiate conditions leading to SCD from athlete’s heart, individuals with LV wall thickening on imaging tests (>11–12 mm)should undergo further diagnosis. According to guidelines features such as abnormal repolarization in the electrocardiogram (ECG) and no changes in wall thickness after antihypertensive therapy suggest that HCM is more probable than hidden hypertension. Important informations are provided also by family history and late gadolinium enhancement (LGE) on cardiac magnetic resonance (CMR). In case of uncertain diagnosis and classification to the “grey zone”, Holter-ECG, ABPM and exercise test should be performed to define disease and to evaluate the risk [37]. Finally, endomyocardial biopsy may be considered [38].

### 3.4 Arrhythmia

Rhythm disturbances are direct cause of death associated with exercise, of which the most dangerous are ventricular arrhythmias. They could be caused by structural heart diseases including HCM, DCM or ACM, adverse myocardial remodeling due to volume overload or increased afterload in hypertension and fibrosis. These findings can be detected during imaging tests. Electrocardiogram can reveal some characteristic features for each cardiomyopathy, but it is essential to confirm it in echocardiography and next in CMR. Suspected cardiomyopathy as well as history of rhythm disturbances is an appropriate reason to perform 24-hour Holter ECG to assess the severity and the type of arrhythmia. Some rhythm disturbances intensify during physical effort, therefore ECG-exercise stress test is necessary to reveal their presence and adequately qualify the patient for the effort load. In individuals with normal cardiac morphology, arrhythmias can be triggered by electrolyte disturbances, to which athletes are particularly exposed during long efforts at high temperature. Additionally, the elderly are characterized by a lower thermoregulation capacity [39]. Therefore adequate hydration and the supply of electrolytes and nutrients during intense exercise are essential, especially in the long endurance competition.

Coronary arteries anomalies and ion channels disorders such as long QT syndrome, short QT syndrome, Brugada syndrome and catecholaminergic polymorphic ventricular tachycardia can be recognized in ECG, 24-hour Holter ECG and exercise testing and are indisputable causes of rhythm disturbances, however, these diagnoses are more likely at age <35 years old than in master athletes.

More common for athletes are supraventricular arrhythmias. There is a strong correlation between hypertension and atrial fibrillation (AF). High blood pressure not only increases probability of AF occurrence, but these both entities share similar risk factors such as age, male gender, genetic predispositions, inflammatory processes, oxidative stress and obesity [40]. Renin hypertension dominates in the majority of hypertensive patients, what is connected with disturbances in renin-angiotensin-aldosterone system. Increased level of aldosterone can result in arrhythmogenic fibrosis of the left atrium [20, 41]. Furthermore, higher blood pressure as a consequence of endothelial dysfunction due to atherosclerosis increases arterial stiffness and is associated with larger left atrial (LA) diameter [42]. LA overload occurs also because of the impaired left ventricular diastolic function. The atrial size has been shown to be proportionally associated with a stroke risk [43]. Enlargement of the left atrium is a common phenomenon in athlete’s heart, but only if accompanied by improvement of atrial emptying due to improved LV diastolic function [44]. Overall the risk of atrial fibrillation is higher and occurs more often in individuals with higher time and intensity of exercises per week [45]. Because this arrhythmia can lead to hemodynamic disturbances, it should be properly treated. Recommended strategy in athletes with atrial fibrillation is ablation [46, 47]. Beta-blockers are often not used due to reduction of maximum heart rate, what is undesirable in endurance sports. Some rhythm disturbances intensify during physical effort, therefore ECG-exercise stress tests is necessary to reveal their presence and adequately qualify the patient for the effort load.

### 3.5 Aortic Disorders

Hemodynamic load may contribute to subtle enlargement of aorta, which is a normal finding in athlete’s heart especially in endurance sports with high dynamic component [48]. However, when combined with hypertension, it has even greater impact on aorta diameter and increases risk of aorta dilatation, which is also associated with aortic stiffness and may result in adverse cardiovascular events [49, 50]. Ultimately high blood pressure with other risk factors or genetic predispositions can lead to aortic dissection, which is one of the causes of SCD [42]. Aortopathies apply even more to veteran athletes, as the elasticity of arteries decreases with age.

## 4. Methods of Assessing Cardiovascular System

**Fig. 2.**
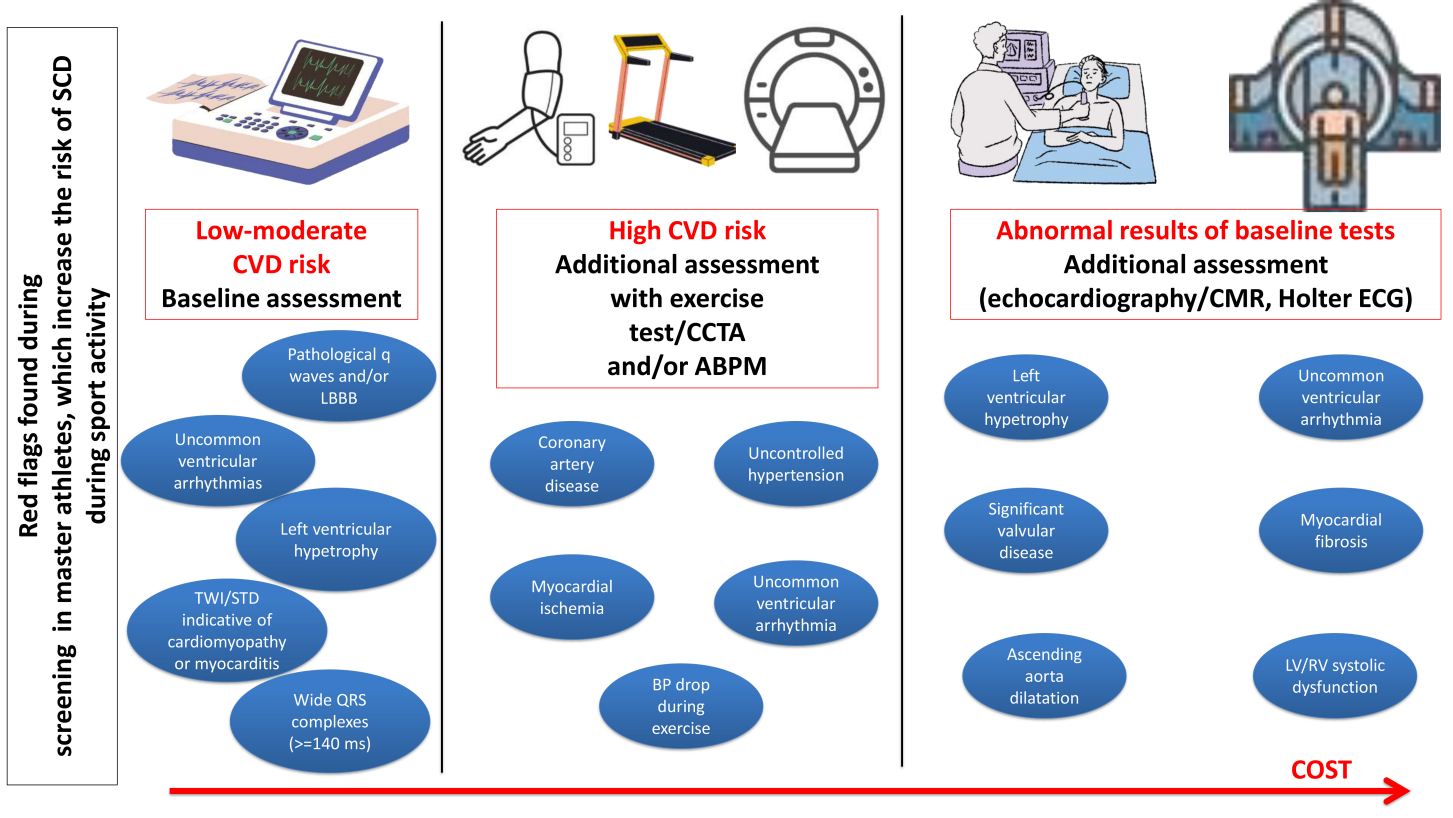
**Potential red flags found during testing of master athletes, which increase the risk of SCD during sport activity**. ABPM, ambulatory blood pressure monitoring; BP, blood pressure; CMR, cardiac magnetic resonance; CVD, cardiovascular disease; CCTA, coronary computed tomography angiography, LBBB, left bundle branch block; STD, ST-segment depression; TWI, T-wave inversion. CVD risk according to SCORE2 calculator.

Before participating in competitive sports it is recommended to evaluate the cardiovascular system in order to identify people with symptoms caused by exercise. Thorough medical history with assessment of cardiovascular risk factors followed by physical examination including heart auscultation and blood pressure assessment should always be the first step. Coronary artery disease is characterized by symptoms appearing during increased effort load, so the resting ECG may not detect abnormalities. However, ECG is a good method to reveal signs of left ventricular hypertrophy, left atrial enlargement, arrhythmia or prior myocardial infarction in this group of athletes. ECG exercise test is the most accessible method to assess athletes. It is excellent to reveal arrhythmia and its changes on exercise. Unfortunately, ECG exercise test has low sensitivity in CAD detection (68%) and ST changes during examination are difficult to assess when intraventricular conduction disturbances are present [51, 52]. Therefore, echocardiographic exercise test is recommended to detect CAD and with 85% sensitivity and similar specificity reveal contractility disturbances, which is associated mainly with ischemic effect [53]. As mentioned earlier mildly decreased left ventricular ejection fraction (LVEF) at rest in healthy athletes should also return to normal during exercise test [36, 53]. Despite that, electrocardiographic exercise test is most often used as a first step to evaluate athletes, because this is a fast, easily accessible method, the result is not so operator-dependent as echocardiography. Stress test has limited effectiveness in detecting mild to moderate CAD and single-vessel disease [54]. More sensitive noninvasive method to detect or exclude CAD is an anatomic evaluation with coronary computed tomography angiography (CCTA), which has high negative predictive value [55]. However, hemodynamic significance is showed only by stress tests [56]. The most precise way to assess cardiac structure, function of chambers and myocardial perfusion is CMR. Additionally, CMR stress perfusion has 90% sensitivity and 94% specificity in CAD detection [57]. CMR and CT are not recommended in patients with advanced renal failure, when assessment of myocardial perfusion is possible with single photon emission computed tomography (SPECT) with similar effectiveness. The final invasive method is coronary angiography, during which, if necessary, coronary angioplasty can be performed. Potential red flags found during testing of master athletes, which increase the risk of SCD during sport activity are presented in Fig. 2. Table 1 presents the differentiation of the athlete’s heart from the effects of hypertension and hypertrophic cardiomyopathy using additional tests.

**Table 1.** **Differentiation of the athlete’s heart from the effect of hypertension and hypertrophic cardiomyopathy**.

	Athlete’s heart	HHD	HCM
Symptoms (chest pain, dyspnoea, palpitations, presyncope/syncope)	–	+	++
Family history of CVD (hypertension or HCM/SCD)	–	+	+
ECG changes:			
- amplitude criteria of LVH	+/–	+	+
- signs of LAE	+/–	+	+
- TWI in infero-lateral leads	–	+	++
- left axis deviation	–	+/–	+/–
- LBBB	–	+/–	+/–
Echocardiography:			
- mild symmetrical LVH	+/–	+	+
- asymmetrical LVH	–	–	++
- regression of LVH after detraining or treatment	+	+	–
- LVOT or intraventricular obstruction	–	+/–	+/–
- diastolic dysfunction	–	+	+
- systolic dysfunction	–	+/–	+/–
- aortic dilatation	–	+/–	–
- balanced heart chamber enlargement	+	–	–
CMR:			
- patchy LGE other than in junction point	–	+/–	+/–
Exercise test:			
- signs of myocardial ischemia	–	+/–	+/–
- BP drop during exercise	–	+/–	+/–
- exaggerated BP response to exercise	–	+	–
- uncommon ventricular arrhythmia	–	+	++
- lower than predicted physical capacity	–	+/–	+/–
Holter ECG:			
- uncommon ventricular arrhythmia	–	+	++

CVD, cardiovascular disease; HCM, hypertrophic cardiomyopathy; SCD, sudden cardiac death; ECG, electrocardiogram; LAE, left atrial enlargement; TWI, T-wave inversion; LBBB, left bundle branch block; LVH, left ventricular hypertrophy; LVOT, left ventricular outflow tract; CMR, cardiac magnetic resonance; LGE, left gadolinium enhancement; BP, blood pressure.

Middle-aged male athletes have higher risk of atherosclerotic plaque formation, which is correlated with increasing training intensity. These plaques are mostly calcified, so their composition does not in general pose a high risk of an undesirable cardiovascular events [58]. This explains why endurance athletes live longer despite the calcification of the vessels [59, 60]. For this reason coronary calcium score may have a limited prognostic value in that group and reference values for coronary calcium score in master athletes are lacking.

Any severe rhythm disturbances in resting ECG, during ECG exercise test or symptoms of arrhythmia in medical history require verification in 24-hour Holter ECG. It is also a tool to assess prognosis in cardiomyopathies or after cardiac adverse remodeling and helps to make a proper decision about possible sports restrictions.

## 5. Screening of Asymptomatic Athletes over 35 Years Old

**Fig. 3.**
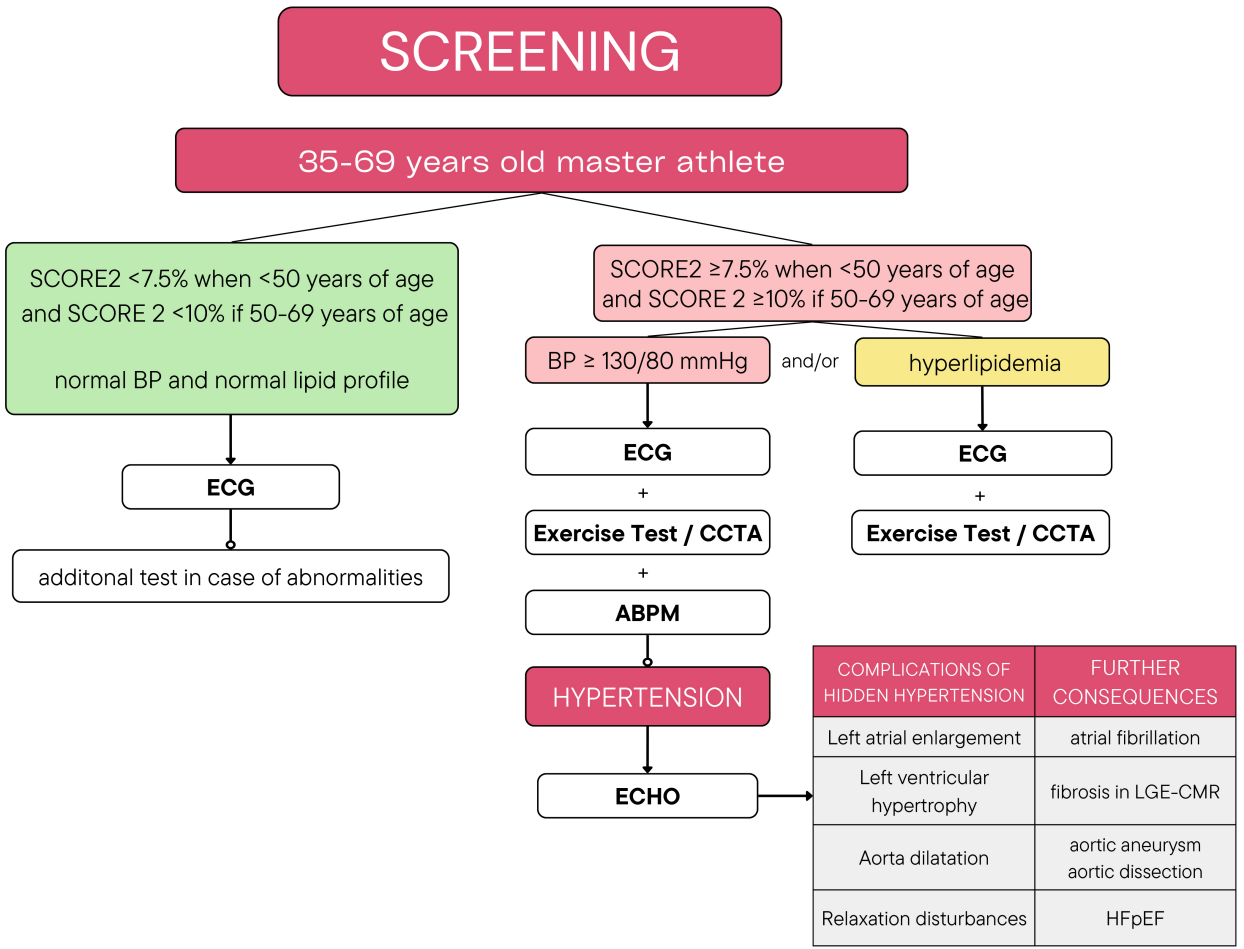
**The proposed scheme for the detection of hidden-hypertension in athletes aged 36–69**. ECG, electrocardiogram; ECHO, echocardiography; CCTA, cardiac computed tomography angiography; ABPM, ambulatory blood pressure monitoring; HFpEF, heart failure with preserved ejection fraction; LGE-CMR, late gadolinium enhancement-cardiac magnetic resonance.

In high performance athlete cardiac adaptation to effort must be differentiated from diseases, which require some restrictions in competitive sport and proper treatment. It is important to take activesteps not only in early detection of coronary artery disease in master athletes stressed by current sports cardiology guidelines [19], but also overt and hidden hypertension before its consequences such as coronary artery disease, rhythm disturbances, aortic disorders and cardiac remodeling with fibrosis occur (Fig. 3). Every individual over 35 years old should have assessment of complete lipid profile, blood pressure at rest and subsequently cardiovascular risk estimated using a SCORE2 scale which includes age, gender, blood pressure, smoking nicotine and non-HDL cholesterol to predict probability of fatal and non-fatal cardiovascular events in 10 years in people from four risk regions in Europe. Some additional factors such as diabetes mellitus, familial hypercholesterolemia, highly increased values of total cholesterol, LDL-cholesterol and blood pressure, chronic kidney disease, previous ACS, diagnosed CCS, past stroke, transient ischemic attack and peripheral atherosclerosis impact on the determination of CVD risk directly and divide patients from low risk to very high risk.

Patients with SCORE2 <7.5% when <50 years of age and <10% if 50–69 years of age, with normal blood pressure and lipid levels in reference values have low or moderate risk of cardiovascular disease. ECG at rest should be taken in this group to assess the rhythm, atrio-ventricular conduction, recognize past myocardial infarction or cardiac remodeling as a part of athlete’s heart. However, because of low sensitivity, the lack of changes in ECG may not always exclude the presence of hypertrophy and sometimes a verification in echocardiography may be needed [61]. All competitive athletes should have control ECG, reassessment of cardiovascular risk at least once a year and several times in year take home BP measurements. According to the guidelines athletes at high risk of CVD with SCORE 2 ≥7.5/10% respectively and additional risk factors of CAD should undergo exercise test or CCTA and then with positive results, coronary angiography is indicated [19, 62].

Hidden hypertension should be suspected in patients who have blood pressure >130/80 mmHg measured twice during examination at rest. High-normal BP at rest in master athletes is associated with complications of hypertension such as greater thickness of LV especially when accompanied with stronger BP response to effort [63]. Therefore in these individuals ABMP is required for further diagnosis. They should have also exercise test or CCTA to exclude CVD which is the most common cause of SCD. In newly recognized arterial hypertension confirmed in ABPM it is necessary to assess heart damage with echocardiography and evaluate aorta, left atrium and left ventricle hypertrophy. Left ventricular hypertrophy with increased wall thickness of 13–15 mm and relaxation disturbances could be an effect of hidden or already diagnosed but improperly treated hypertension or not revealed previously HCM. Impairment of diastolic function in tissue doppler echocardiography accompanied with symptoms of biventricular heart failure requires differentiation with restrictive cardiomyopathy (RCM). CMR may be needed to further investigate the heart, obtain more accurate measurements, revealed late gadolinium enhancement areas and make the right decision on eventual restrictions in sports [64]. Hypertension requires treatment with lifestyle changes or already pharmacotherapy when BP is >160/100 mmHg. Athletes with diagnosed hypertension with cardiac complications like LVH or uncontrolled disease with blood pressure >160/100 mmHg should suspend participation in competitive sport until they obtain correct values. Blood pressure under control and no organ damages is sufficient for admission to competitive sports.

## 6. Prevention

Despite the important role of screening, an essential method to prevent acquired cardiovascular diseases is prevention. Most of problems related to the cardiovascular system as well as hidden hypertension are caused by unhealthy lifestyle and it usually concerns recreational older athletes more than younger professionals, which have special diets and training plan. Considering that age is also a risk factor of hypertension and CVD due to changes in the structure of arteries [65], master athletes should be especially careful about other factors, as a sedentary lifestyle is only one of them [16, 62]. Strong correlation between dietary total antioxidant capacity and reduced cardiovascular risk should contribute to changes in eating habits not only in people with risk factors, but in all those who want to maintain a healthy lifestyle [66]. Additionally, nonsteroidal anti-inflammatory drugs, which are very often used during sports injuries or some inconsistent with the principles of “fair play” substances, prohibited by World Anti-Doping Agency such as erythropoietin anabolic steroids are characterized by increasing blood pressure [67, 68, 69]. Raising awareness of the risk factors is essential in preventing an increasing number of hypertensive patients.

## 7. Summary

Sudden cardiac death is very disturbing phenomenon and still appearing during sports competitions, despite of evolving screening methods. On the other side, it is important to cooperate properly with such a specific patient as master athlete and to raise awareness of need for medical examination before performing extreme exercises. The current guidelines in sports cardiology are aimed at revealing CAD, but likewise hidden hypertension may pose a risk of cardiovascular event. The overlapping effects of high blood pressure and athlete’s adaptation to chronic intensive effort, especially mild left ventricular hypertrophy, may cause diagnostic difficulties. Regular physical activity reduces resting blood pressure, what makes hypertension even more difficult to detect, therefore the proposed screening scheme facilitates the search of athletes from high-risk group.
